# Evolution of Codon Usage Bias in Henipaviruses Is Governed by Natural Selection and Is Host-Specific

**DOI:** 10.3390/v10110604

**Published:** 2018-11-01

**Authors:** Naveen Kumar, Diwakar D. Kulkarni, Benhur Lee, Rahul Kaushik, Sandeep Bhatia, Richa Sood, Atul Kumar Pateriya, Sushant Bhat, Vijendra Pal Singh

**Affiliations:** 1 National Institute of High Security Animal Diseases, Bhopal 462022, India; ddkulkar@gmail.com (D.D.K.); sbhatia1967@gmail.com (S.B.); richa.bhatia0609@gmail.com (R.S.); aatulpateriya@gmail.com (A.K.P.); vijendra61@gmail.com (V.P.S.); 2 Department of Microbiology, Icahn School of Medicine at Mount Sinai, New York, NY 10029, USA; benhur.lee@mssm.edu; 3 Supercomputing Facility for Bioinformatics & Computational Biology, Indian Institute of Technology, Delhi 110016, India; rahul.hau.bioinfo@gmail.com or rahul.kaushik@riken.jp; 4 Laboratory for Structural Bioinformatics, Center for Biosystems Dynamics Research, RIKEN, Kanagawa 2300045, Japan; 5 The Pirbright Institute, Woking GU24 0NF, UK; sushant.shanty@gmail.com

**Keywords:** Henipaviruses, codon usage bias, host adaptation, natural selection, evolution

## Abstract

Hendra virus (HeV) and Nipah virus (NiV) are among a group of emerging *bat*-borne paramyxoviruses that have crossed their species-barrier several times by infecting several hosts with a high fatality rate in *human beings*. Despite the fatal nature of their infection, a comprehensive study to explore their evolution and adaptation in different hosts is lacking. A study of codon usage patterns in henipaviruses may provide some fruitful insight into their evolutionary processes of synonymous codon usage and host-adapted evolution. Here, we performed a systematic evolutionary and codon usage bias analysis of henipaviruses. We found a low codon usage bias in the coding sequences of henipaviruses and that natural selection, mutation pressure, and nucleotide compositions shapes the codon usage patterns of henipaviruses, with natural selection being more important than the others. Also, henipaviruses showed the highest level of adaptation to *bats* of the genus *Pteropus* in the codon adaptation index (CAI), relative to the codon de-optimization index (RCDI), and similarity index (SiD) analyses. Furthermore, a comparison to recently identified henipa-like viruses indicated a high tRNA adaptation index of henipaviruses for *human beings*, mainly due to F, G and L proteins. Consequently, the study concedes the substantial emergence of henipaviruses in *human beings*, particularly when paired with frequent exposure to direct/indirect *bat* excretions.

## 1. Introduction

Henipaviruses, especially Hendra virus (HeV) and Nipah virus (NiV), are reckoned to be among the deadliest viruses for which *bat* has been implicated as a natural reservoir host. These viruses are prototypical members of the genus *Henipavirus* and family *Paramyxoviridae* (subfamily–*Paramyxovirinae*) [1]. Recently, Cedar virus (CedPV) has been included to the *Henipavirus* genus [2] which is also *bat*-borne, but is yet to be linked to any clinical disease. Certain unique features of HeV and NiV distinguish them from the other members of the *Paramyxoviridae* family, which include bigger genome size (18,234 and 18,246 nt, respectively, while rest member’s genome size varies from 15–16 kbps), broad host range (*bat*, *pig*, *horse*, *human*, and *dog*), high virulence and zoonotic potential [3,4]. Their genome is composed of single-stranded, non-segmented, and negative-sense RNA. Henipaviruses comprise of six transcription gene units encoding six major structural proteins, namely the nucleocapsid protein (N), phosphoprotein (P), matrix protein (M), fusion protein (F), glycoprotein (G) and large protein (L) or RNA polymerase, in the order 3′-N-P-M-F-G-L-5′. Also, three predicted non-structural proteins are reported in henipaviruses, namely C, V and W [4]. The V and W proteins are expressed by insertion of a single or two non-templated G residues, respectively, at the editing site within the P gene, while the C protein is expressed from an alternative open reading frame (ORF) [4].

Henipaviruses are the only currently recognized zoonotic paramyxoviruses capable of causing severe infection in a broad range of animals and are fatal to both *human beings* and animals [5]. To date, HeV has caused 60 outbreaks resulting in the death of 102 *horses*, four fatalities of seven *human* cases and two HeV seropositive *dogs*, all arising in the northeastern coastal region of Australia [6]. Approximately ~620 cases of *human* NiV infection with 322 fatalities have been reported so far [7]. *Fruit Bats* (flying foxes or megabats) within the suborder *Megachiroptera*, predominantly those of the genus *Pteropus*, have been identified as the major natural reservoir host of henipaviruses [2,8,9,10]. However, the topographical distribution of these reservoir *bats* partly corresponds with the distribution of henipaviruses outbreaks or spillover events. Furthermore, the evidence for the presence of henipaviruses in a wide variety of other *bat* species within the *Megachiroptera* and *Microchiroptera* suborders is ever growing [11,12,13,14].

The inherent redundancy of a universal genetic code allows the translation of 61 sense codons into 20 different amino acids. Thus, most amino acids are encoded by several synonymous codons (codons coding for the same amino acid). The synonymous codons are not used arbitrarily and, usually, one codon is used more frequently than others. This biased use of codons has been observed in all branches of life and results in species-specific codon usage bias [15,16]. The evolution of synonymous codons usage has been associated frequently with two major factors namely directional mutation pressure and natural selection. The directional mutation pressure explains the interspecific difference in the complete genome sequence, which is predominantly governed by the biased usage of AU/GC content [17]. However, natural selection basically involves a selection acting on a specific subset of codons (most preferred codons to match the host tRNAs abundance or translation selection) or against a sequence pattern (CpG) that activates innate immunity (Toll-like receptor 9) or against a target of immunity effectors (UpA dinucleotide, targeted by RNAse L) [18,19,20,21,22]. However, several other factors such as secondary RNA structure, regulatory structural RNA elements, and even viral RNA packaging also influence the codon usage bias [23,24,25].

The dependence of viruses on the host cellular machinery for various key processes viz. replication, protein synthesis, and transmission, reflect that the overall viral fitness, survival and evolution are likely to be dictated by the interaction between the codon usage of the virus and that of its host [26]. Considering this, the information about the codon usage of viruses could provide an insight into host-adapted evolution, factors driving the codon usage bias, and regulation of genes expression. Consequently, in this study, we employed a broad range of methods to investigate (i) the key factors responsible for the codon usage bias of henipaviruses; (ii) contribution of synonymous codon usage in the evolutionary processes of henipaviruses; and (iii) the fitness of henipaviruses to various hosts.

## 2. Materials and Methods

### 2.1. Sequence Data Analyzed

The complete coding genomic sequences of 13 isolates of NiV and HeV reported across the world to date, were obtained from the National Center for Biotechnological Information (NCBI) (available at https://www.ncbi.nlm.nih.gov/) and the Virus Pathogen Resource database (available at https://www.viprbrc.org/brc/home.spg?decorator=vipr), and accessed as on 14 December 2017. For each strain, open reading frames (ORFs) were concatenated in the following order (N + P + M + F + G + L). The demographics of each strain are provided in the Supplementary Table S1.

### 2.2. Phylogenetic Analysis

Phylogenetic reconstruction was inferred using the Maximum Likelihood statistical method with TN93 + G substitution model implemented in the MEGA 7 [27]. The bootstrap analyses of the trees were performed with 1000 replicates of dataset to determine the robustness of the individual nodes of the tree. The scale bar indicates nucleotide substitutions per site. For each strain, the following data set is furnished: Virus/Species affected/Country/strain name/year of isolation/GenBank accession numbers.

### 2.3. Nucleotide Composition Analysis

The diverse nucleotide compositional properties were calculated for the coding sequences of HeV and NiV genomes. These compositional properties comprise the frequencies of occurrence of each nucleotide (A%, U%, G%, and C%); AU and GC contents; each nucleotide at the third position of the synonymous codons (A3%, U3%, G3%, and C3%); nucleotides G + C at the first (GC1), second (GC2), and third codon positions (GC3); mean frequencies of nucleotides G + C at the first and the second positions (GC12). The codons for Met (AUG), Trp (UGG) and termination codons (UAA, UGA, UAG) are unlikely to contribute in the codon usage bias, and therefore, these were excluded from the analysis.

### 2.4. Relative Synonymous Codon Usage Analysis

The relative synonymous codon usage (RSCU) value of a codon is better defined as the ratio of its observed frequency to its expected frequency providing that all the codons encoding a particular amino acid are used equally [28]. The RSCU values for all the coding sequences of the HeV and NiV genomes were calculated to determine the synonymous codon usage patterns without the confounding influence of amino acid compositions or sequence length. The RSCU values were estimated as follows [28]:

$$RSCU=\frac{g_{ij}}{\sum_{j}^{n_{i}}g_{ij}}n_{i}\;$$

where *g_ij_* is the observed number of the *i*th codon for the *j*th amino acid, which has *n_i_* kinds of the synonymous codons. The synonymous codons with the RSCU values of <1.0, 1.0 and >1.0 represent negative codon usage bias (less abundant codons), no bias (equal usage of all the synonymous codons) and positive codon usage bias (abundant codons), respectively.

### 2.5. Effective Number of Codons Analysis

An effective number of codons (ENc) analysis of HeV and NiV coding sequences were performed to quantify the absolute codon usage bias using the formulae [29]:

$$ENc=2+\frac{9}{{\bar{F}}_{2}}+\frac{1}{{\bar{F}}_{3}}+\;\frac{5}{{\bar{F}}_{4}}+\frac{3}{{\bar{F}}_{6}}\;$$

where $\bar{F}$(*i* = 2, 3, 4, 6) is the mean of $\bar{F_{i}}$ values for the *i*-fold degenerate amino acid. The $\bar{F_{i}}$ values were calculated using the formulae:

$$\bar{F_{i}}=\frac{n\sum_{j=1}^{i}{\left(\frac{n_{j}}{n}\right)}^{2}-1}{n-1}\;$$

where *n* is the total number of occurrences of the codons for that amino acid and *n_j_* is the total number of occurrences of the *j*th codon for that amino acid.

The ENc values range from 20 to 61 [29]. The ENc value of 20 states an extreme codon usage bias (only one of the possible synonymous codons is used for the corresponding amino acid); while that of 61 states no bias at all (all possible synonymous codons are used equally for the corresponding amino acid). Consequently, the lower the ENc value, the higher the extent of codon usage bias.

### 2.6. ENc–GC3s Plot Analysis

An ENc–GC3s plot offers qualitative information about the role of directional mutation pressure in the codon usage bias. Herein, the ENc values are the ordinate and the GC3s values (frequency of either a guanine or cytosine at the third codon position of the synonymous codons, excluding Met, Trp, and stop codons) are the abscissa [29]. The codon usage is constrained only by G + C mutation bias when the predicted ENc values are scoring at or around the standard curve (functional relation between expected ENc and GC3s). Else, the other factors such as natural selection, RNA folding and genetic drift play a significant role in shaping the codon usage bias. Expected ENc values were calculated as follows:

$$ENc_{expected}=2+s+\frac{29}{s^{2}+\left(1-s^{2}\right)}\;$$

where *s* is the frequency of G + C at the third codon position of synonymous codons (i.e., GC3s).

### 2.7. Parity Rule 2 Analysis

The Parity rule 2 (PR2) plot is an alternative way to assess the qualitative effects of directional mutation pressure and the natural selection on the codon usage bias. In the PR2 plot, the AU-bias [A3/(A3 + U3)] at the third codon position of the four-codon amino acids of entire coding sequences is the ordinate, and the GC-bias [G3/(G3 + C3)] is the abscissa. The center of the plot, where both coordinates cross at 0.5, denotes no bias between the influence of the mutation pressure and natural selection [30].

### 2.8. Neutrality Plot Analysis

The mutation pressure in the evolution of synonymous codon usage has shown a directionally towards a higher or lower GC content of the genomes and these directional changes have been seen more in neutral parts of the genome. Since GC content at the third codon position (GC3) represents one of the most neutral nucleotides in the genome, it becomes a more important contributor in the directional mutation pressure. The neutrality plot (GC12 vs. GC3) evaluates the relationship among the three codon positions to reflect the role of directional mutation pressure. Therefore, the degree to which directional mutation pressure and the natural selection influenced the codon usage patterns of the HeV and NiV coding sequences was estimated via neutrality plots. The neutrality plot was drawn with GC12 as ordinate and GC3 as abscissa and each dot represents an independent HeV/NiV strain. In this plot, the slopes of the regression lines indicate the evolutionary rates of the directional mutation pressure-natural selection equilibrium. In a plot regression, a zero slope (all the points positioned on the parallel lines of the abscissa) indicates no effect of directional mutation pressure, while a slope of one (the points positioned on the diagonal line) is indicative of complete neutrality [17].

### 2.9. Codon Adaptation Index

Codon adaptation index (CAI) is used to quantify the codon usage similarities between the virus and host coding sequences, and is likely to indicate an approximation of the success of the virus/heterologous gene expression in the host. The index values range from 0 to 1, where the score 1 represents the tendency of a gene to always use the most frequently used synonymous codons in the host [31]. The CAI analysis of the HeV and NiV coding sequences were performed using the CAIcal web-server (available at: http://genomes.urv.es/CAIcal/) [32]. The synonymous codon usage data of a *human* (*Homo sapiens*), *pig* (*Sus scrofa*), *horse* (*Equus caballus*), *dog* (*Canis familiaris*) and *cat* (*Felis catus*) were retrieved from the codon usage database (available at: http://www.kazusa.or.jp/codon/) [33]. The complete genomes of *Pteropus alecto* (ASM32557v1), *Pteropus vampyrus* (GCF_000151845.1), *Myotis brandtii* (ASM41265v1), *Myotis lucifugus* (GCF_000147115.1), *Myotis davidii* (ASM32734v1), and *Eptesicus fuscus* (GCA_000308155.1) were downloaded from The National Center for Biotechnology Information (available at: https://www.ncbi.nlm.nih.gov).

### 2.10. tRNA Adaptation Index

The tRNA adaptation index (tAI) is a widely accepted parameter to measure the efficiency by which a coding sequence is recognized by the intra-cellular tRNA pool of a host with a better correlation with protein abundance [34]. A new approach which does not require gene expression measurements of test organism is utilized, and it calculates species-specific tAI wobble weights by optimizing the correlation between the tAI and a measure of codon usage bias [35]. The absolute adaptiveness value of the *i*^th^ codon was calculated using the following equation:

$$W_{i}=\sum_{j=1}^{n_{i}}\left(1-S_{ij}\right)\cdot tGCN_{ij}\;$$

where *n_i_* is the number of tRNA isoacceptors that recognize the *i*th codon; *tGCN_ij_* is the gene copy number of the *j*th tRNA that recognizes the *i*th codon; and *S_ij_* is a selective constraint on the efficiency of the interaction between the *i*th codon and *j*th tRNA [36]. The codon relative adaptiveness value (*w_i_*) was obtained by dividing each *W_i_* with the maximum *W_i_* value over all codons [36]. The tAI of a gene is defined as the geometric mean of the *w_i_* values of its codons. The frequencies of tRNAs of the studied host species were obtained from the GtRNAdb database (available at: http://gtrnadb.ucsc.edu/) [37].

### 2.11. Relative Codon Deoptimization Index

The trends in the relative codon deoptimization index (RCDI) of HeV and NiV coding sequences with respect to the host species were evaluated by comparing the similarity in codon usage of HeV and NiV coding sequences with that of the reference genomes of host species. The RCDI values were computed using a web-based RCDI/eRCDI server (available at: http://genomes.urv.es/CAIcal/RCDI/) [38]. This server calculates the RCDI by the following equation:

$$RCDI=\sum_{i=1}^{61}\frac{\frac{CiFa}{CiFh}\;N_{i}}{N}\;$$

where *CiFa* is the relative frequency of codon *i* for a specific amino acid in the test sequence; *CiFh* is the relative frequency of codon *i* for a specific amino acid in the reference genome sequence; *N_i_* is the number of occurrences of codon *i* in the test sequence; and *N* is the total number of codons in the test sequence. The RCDI value of 1 specifies that the virus shows a complete host-adapted codon usage pattern; however, RCDI values greater than 1 are indicative of deoptimization of the codon usage patterns of the virus from that of its host [38,39].

### 2.12. Similarity Index

The similarity index, *D*(*A*,*B*), provides an insight into the overall influence of the codon usage pattern of the host on the codon usage of the virus, and was calculated as follows:

$$R\left(A,B\right)=\frac{\sum_{i=1}^{59}a_{i}\times b_{i}}{\sqrt{\sum_{i=1}^{59}a_{i}{}^{2}\times \sum_{i=1}^{59}b_{i}{}^{2}}}\;$$

$$D\left(A,B\right)=\frac{1-R\left(A,B\right)}{2}\;$$

where *R*(*A*,*B*) is defined as the cosine value of the angle included between *A* and *B* spatial vectors, and represents a degree of similarity between the virus and host overall codon usage patterns. *a_i_* is defined as the RSCU value for a specific codon among the 59 synonymous codons of the virus coding sequence. *b_i_* is the RSCU value for the same codon in the host. The *D*(*A*,*B*) value ranges from 0 to 1.0 [40]. The higher *D*(*A*,*B*) means a stronger influence of environment-related synonymous codon usage patterns of hosts to that of viruses.

### 2.13. Statistical Analysis

Correspondence analysis (COA), a multivariate statistical method is widely implemented for studying the trends in codon usage variations [41]. In this analysis, the degrees of freedom were condensed to 40 (from 59 synonymous codons) by eliminating the variations caused by the unequal usage of amino acids while generating a correspondence analysis of RSCU. The major trends within the data set were estimated based on the measurement of relative inertia, and virus strains were arranged according to their positions along the axes of major inertia. COA was performed on the RSCU values of codons and complete coding sequences of HeV and NiV. In addition, Spearman’s rank correlation and linear regression analyses were performed using XLSTAT Version 2016 and GraphPad Prism 7.01 (GraphPad Software, San Diego, CA, USA).

## 3. Results

### 3.1. HeV and NiV Have Quite Distinct Evolutionary Patterns

**Figure 1:**
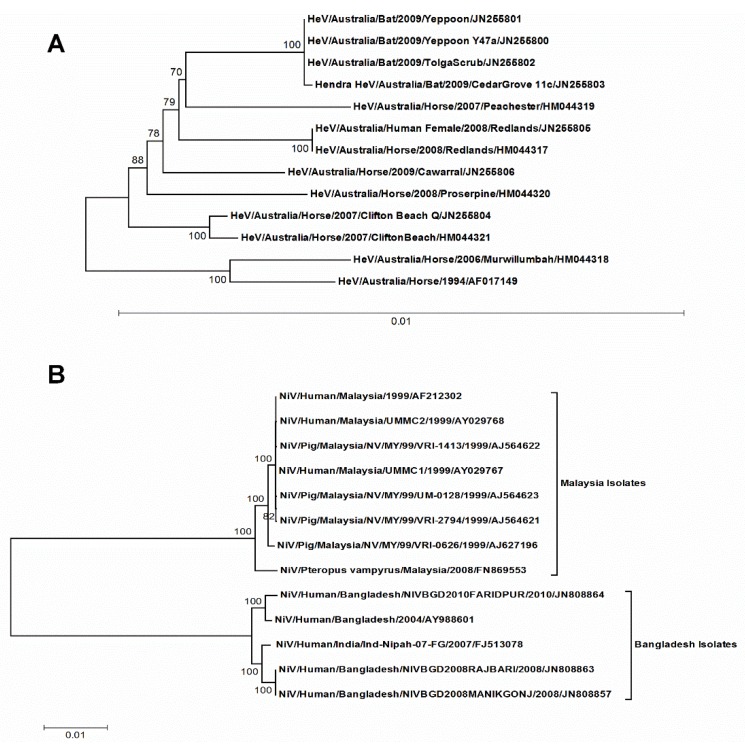
The maximum likelihood trees of HeV (**A**) and NiV (**B**) complete coding genomic sequences. The trees were constructed by TN93 + G substitution model implemented in MEGA software version 7. The reliability of the trees was assessed by bootstrap with 1000 replications. The bootstrap values greater than 70 are shown. Scale bars represent substitutions per site. For each strain, the following data are furnished: Virus/Species affected/Country/strain name/year of isolation/GenBank accession nos. HeV, Hendra virus; NiV, Nipah virus.

The complete coding sequences of HeV and NiV were subjected to phylogenetic analyses and the respective trees were generated using the TN93 + G model. In the case of NiV, two separate clusters were formed where one cluster had all the Malaysian isolates, while the other accommodated the Bangladesh and the Indian isolates (Figure 1B). The isolates in each cluster had >99% nucleotide sequence similarity among them while one cluster had <94% nucleotide sequence similarity with other cluster suggesting two independent geographical introductions for these clusters. The case of HeV was quite distinct where all the isolates had >99% nucleotide sequence similarity among them, suggesting a common geographical introduction (Figure 1A).

### 3.2. Trends in Codon Usage Variations of Henipaviruses

**Figure 2:**
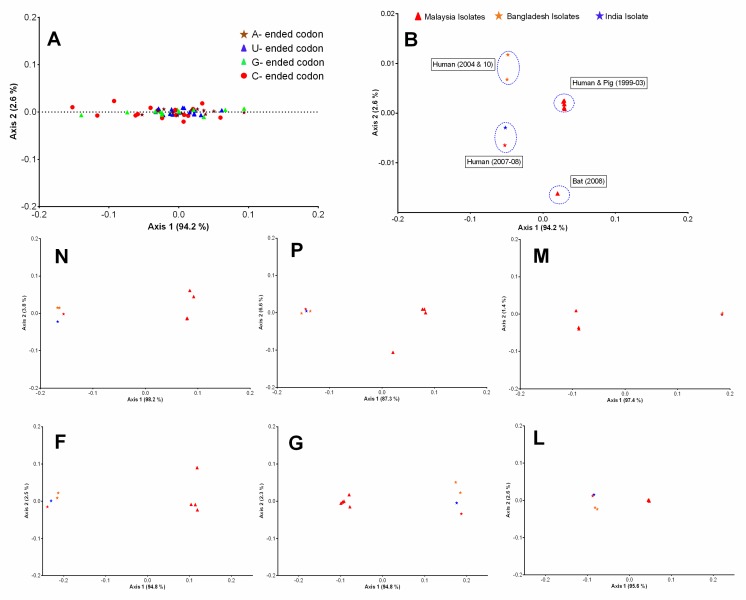
Codon usage variations in the coding sequences of NiV. Correspondence analysis (COA) was performed on the RSCU of codons (**A**) and complete coding sequences of NiV isolates (**B**) and plotted on the first two-main-axes. The COA analysis of individual protein-coding sequences (N, P, M, F, G, L) of NiV was also plotted on the first two-main-axes. The different base-ended codons and geographically distinct NiV isolates were color labeled.

The correspondence analysis (COA) was performed to examine the synonymous codons usage variations among the coding sequences of HeV and NiV. In NiV coding sequences, the analysis was restricted to two principal axes (ƒ′1 and ƒ′2) which accounted for the majority of data inertia (ƒ′1 = 94.2%, ƒ′2 = 2.6%). The COA constructed on the RSCU of codons revealed that codons were frequently distributed along the first (ƒ′1) principal axis with most extreme values occupied by the rarely used codons (primarily the codons ending with a C or G) (Figure 2A). The COA generated on the RSCU of NiV isolates coding sequences formed four well-defined clusters which were phylogenetically and temporally distinct (Figure 2B).

**Figure 3:**
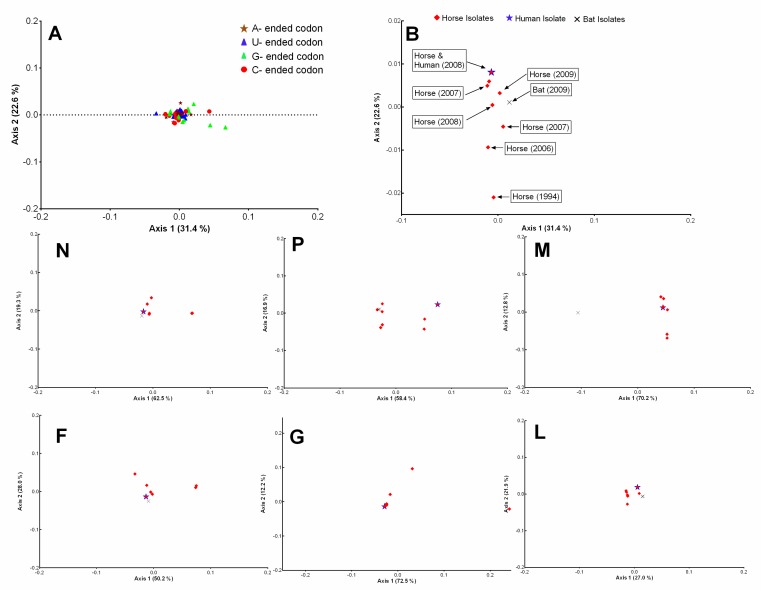
Codon usage variations in the coding sequences of HeV. Correspondence analysis (COA) was performed on the RSCU of codons (**A**) and complete coding sequences of HeV isolates (**B**) and plotted on the first two-main-axes. The COA analysis of different protein-coding sequences (N, P, M, F, G, L) of HeV was also plotted on the first two-main-axes. The different base-ended codons and host-wise HeV isolates were color labeled.

In the case of HeV, the first two principal axes (ƒ′1 and ƒ′2) accounted for more than 50% of data inertia (ƒ′1 = 31.4%, ƒ′2 = 22.6%); an additional two axes (ƒ′3 = 14.5%, ƒ′4 = 10.9%) were also included in this analysis to examine whether these axes had any correlation with any parameters of codon bias. The majority of the codons lay at the intersection of ƒ′1 and ƒ′2, while a few codons (frequently ending with a C or G; CGG, UCG, CGC, GCG, CGU) were outliers (Figure 3A). The COA generated on the RSCU of HeV isolates coding sequences using ƒ′1 and ƒ′2 displayed more discrete distribution compared to ƒ′3 and ƒ′4. This distribution across ƒ′1 and ƒ′2 did not form well-defined clusters, like that of NiV, however, they were phylogenetically distinct (Figure 3B).

### 3.3. Influence of Nucleotide Compositions on the Codon Usage Bias

The nucleotide compositions of the NiV and HeV genomes were computed to understand the possible influence of compositional constraints on the codon usage and were correlated with the principal axes generated in COA. The mean compositions (%) of nucleotides A (NiV = 33.14 ± 0.11, HeV = 32.61 ± 0.03) were found to be the highest, followed by U (NiV = 26.83 ± 0.05, HeV = 26.61 ± 0.03), G (NiV = 21.25 ± 0.07, HeV = 21.87 ± 0.03), and C being the lowest (NiV = 18.79 ± 0.08, HeV = 18.91 ± 0.04). The similar pattern was also observed for the nucleotides at the third position of synonymous codons (A3, G3, U3, and C3) (Supplementary Table S2).

Besides, correlation of different nucleotide compositions with the principal axes of the COA was also performed. In NiV, axis 1 had a distinct positive correlation with A (*r* = 0.786, *p* = 0.001) and U (*r* = 0.676, *p* = 0.01), and a negative correlation with G (*r* = −0.885, *p* < 0.0001) and C (*r* = −0.692, *p* = 0.008) (Supplementary Table S3). There was a significant positive correlation between ENc and GC3s (*r* = 0.863, *p* < 0.0001), while ENc (*r* = −0.853, *p* < 0.001) and GC3s (*r* = −0.831, *p* < 0.001) had negative correlation with axis 1. The case of HeV was different, being shared among four principal axes. The G (%) and C (%) had a distinct positive correlation with axis 1 (*r* = 0.671, *p* < 0.05) and axis 3 (*r* = 0.607, *p* < 0.05), respectively, while U (%) had a distinct negative correlation with axis 2 (*r* = −0.778, *p* < 0.01) (Supplementary Table S3). There was a significant positive correlation between ENc and GC3s (*r* = 0.821, *p* < 0.01), and both ENc (*r* = 0.717, *p* < 0.05) and GC3s (*r* = 0.637, *p* < 0.05) had a positive correlation with axis 3. These results demonstrated that compositional constraints indeed affects codon usage bias in both the NiV and HeV, albeit, in a different way.

### 3.4. Relative Synonymous Codon Usage (RSCU) Analysis

The virus codon usage patterns are specific to family, genus and even at the species level. In order to analyze this specificity in detail at the species level, the RSCU values of NiV and HeV genomes were computed and compared with their host species. In general, the RSCU values of the majority of the codons scored between 0.6 and 1.6 (Table 1). Interestingly, henipaviruses strongly preferred NNA codons, even in the case of highly suppressed CGN codons of arginine, where CGA is over-represented (RSCU > 1.6) (Table 1). However, the preference of NNA or NNU codons was observed for the codons showing RSCU > 1.6. In the CGN codons group, CGC of HeV and CGG of NiV were highly suppressed. Almost all the RSCU values of less than 0.5 were observed in CGN/NCG codons, indicating a strong CpG deficiency or suppression. This CpG deficiency is generally maintained in RNA viruses to avoid innate immune responses and also to mimic the host’s codon usage as an optimization to the available tRNAs pool. On comparing the RSCU values of 59 sense codons of HeV and NiV genomes with the RSCU values of their host species, we observed that none of the over-represented codons were common among the HeV and NiV genomes with the host species (Table 1). Nevertheless, NCG codons in henipaviruses genomes and their hosts were under-represented. In general, the RSCU values of NiV and HeV showed a similar trend in coding for different amino acids with minor differences.

### 3.5. L Gene of NiV and N Gene of HeV Showed a Comparatively High Codon Bias

**Figure 4:**
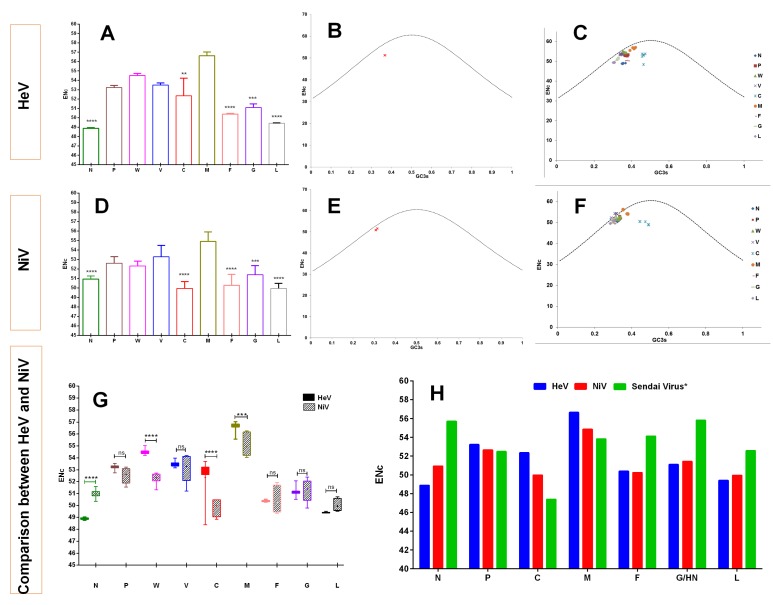
Differential codon bias on the coding sequences of henipaviruses. The gene-wise ENc values for HeV (**A**) and NiV (**D**) are presented. The Kruskal-Wallis test was used to see any evidence of significant differences. The ENc–GC3s plots for the complete coding sequences of HeV (**B**) and NiV (**E**) are shown. The standard curve (functional relations between expected ENc and GC3s), where the codon usage bias was determined by the GC3s composition only is denoted by a dotted line. Similarly, ENc–GC3s plots for the individual protein-coding sequences of HeV (**C**) and NiV (**F**) are also shown. G depicts a comparison of the ENc values among the different proteins encoded by HeV and NiV. The two-way analysis of variance (ANOVA) with Tukey’s honestly significant difference test was used to see any evidence of significant difference. H represents the ENc values comparison of HeV, NiV, and Sendai virus (distantly related). Note: Error bars denote the standard deviation (SD). **** *p* < 0.0001; *** *p* < 0.001; ** *p* < 0.01; ns—non-significant.

The codon bias in the HeV and NiV genomes were estimated through the ENc values. The ENc values were found to be almost similar for HeV (51.21 ± 0.07) and NiV (51.06 ± 0.38). However, the protein-coding genes of both the viruses showed quite distinct codon bias. For instance, N, L, F, G and C of HeV had significantly higher codon usage bias as compared to the least biased M gene (*p* < 0.01–0.0001) (Figure 4A), whilst codon usage bias of C, L, F, N and G of NiV was significantly higher compared to the least biased M gene (*p* < 0.001–0.0001) (Figure 4D). A comparison of individual protein-coding genes of these two viruses revealed that the ENc values of N_HeV_ were significantly lower as compared to N_NiV_ (*p* < 0.0001), while W, C and M of HeV had significantly high ENc values compared to NiV (*p* < 0.001–0.0001) (Figure 4G). A wide range of ENc values detected in the C protein of HeV was due to two isolates (HeV/Human/Australia/Redlands/2008/JN255805 and HeV/Horse/Australia/Redlands/2008/HM044317), probably having the same source of infection.

### 3.6. Mutation Bias Acts Differently on the Protein-Coding Genes of NiV and HeV

The role of directional mutation pressure in governing the codon usage bias in the NiV and HeV coding genomic sequences was investigated by constructing PR2 and ENc–GC3s plots. In the PR2 plot, a preponderance of AU bias in the fourfold degenerate codon families for both the NiV and HeV coding genomic sequences was observed (Supplementary Figure S1).

Furthermore, in the ENc–GC3s plot, all the points corresponding to the HeV and NiV isolates clustered below the standard curve (Figure 4B,E). None of the HeV and NiV isolates fell on the standard curve, which would have indicated the absolute role of directional mutation pressure in codon usage bias, whereas the below-curve clustering is suggestive of the dominant influence of natural selection. However, the influence of directional mutation pressure was not entirely missing and the effects of directional mutation pressure and natural selection on individual protein-coding sequences of both HeV and NiV varied, even within a single isolate (Figure 4B,E). In the case of optimal codon usage, the genes would have lied on or just below the expected curve. Of note, the C protein-coding sequence of both HeV and NiV clustered far below the expected curve, indicating a high codon bias having a significant correlation with gene expression (Figure 4C,F).

### 3.7. Natural Selection Prevails in Shaping the Codon Usage Patterns in Henipaviruses

The ENc–GC3s plot demonstrated that both directional mutation pressure and natural selection have contributed to shaping the codon usage patterns of HeV and NiV. Furthermore, the magnitude of natural selection or directional mutation pressure in influencing the codon usage bias in the coding sequences of both NiV and HeV was investigated by the neutrality plots.

**Figure 5:**
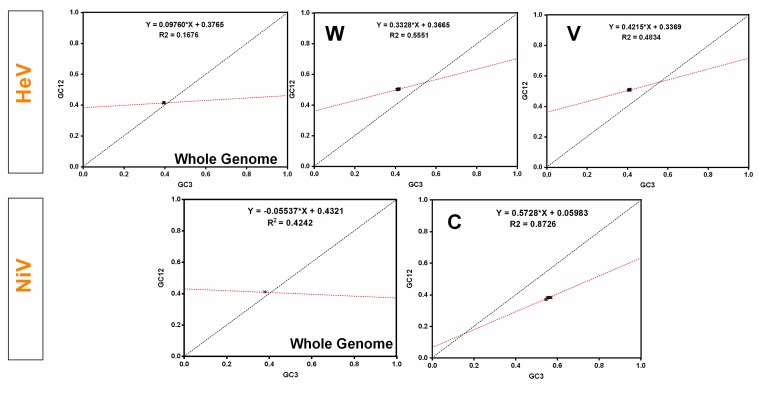
Neutrality plots for the entire coding sequences of HeV and NiV. In neutrality plots, GC12 of different virus isolates were plotted against GC3. The linear regression of GC12 against GC3 is presented by a red-dotted line (HeV—Y = 0.06900*X + 0.3877, *R*^2^ = 0.0339; NiV—Y = −0.05536*X + 0.4321, *R*^2^ = 0.424).

In the neutrality plot analysis, a non-significant correlation between GC12 and GC3 in HeV (*p* > 0.05) and a negative correlation in NiV (*r* = −0.588, *p* < 0.05) was observed. However, the slopes of the regression lines in HeV and NiV were 0.0976 and −0.0553, respectively (Figure 5). This indicates that the relative neutrality (directional mutation pressure) in HeV and NiV was 9.76% and 5.53%, respectively. Thus, the contribution of natural selection in influencing the codon usage patterns was high i.e., 90.24% in HeV and 94.47% in NiV. Furthermore, individual protein sequences of HeV and NiV were subjected to neutrality plots. In the case of NiV, a significant correlation between GC12 and GC3 along with regression slope towards 1 was observed in C protein, which indicates that C protein is under strong directional mutation pressure (57.28%) as compared to that of natural selection (Figure 5 and Supplementary Table S4). Similarly, W (33.28%) and V (42.15%) proteins of HeV also experienced high directional mutation pressure. Overall, the influence of the natural selection remained predominant in shaping the codon usage patterns in the complete coding sequences of both HeV and NiV.

### 3.8. HeV and NiV Showed Host-Specific Discrete Codon Adaptation Patterns

**Figure 6:**
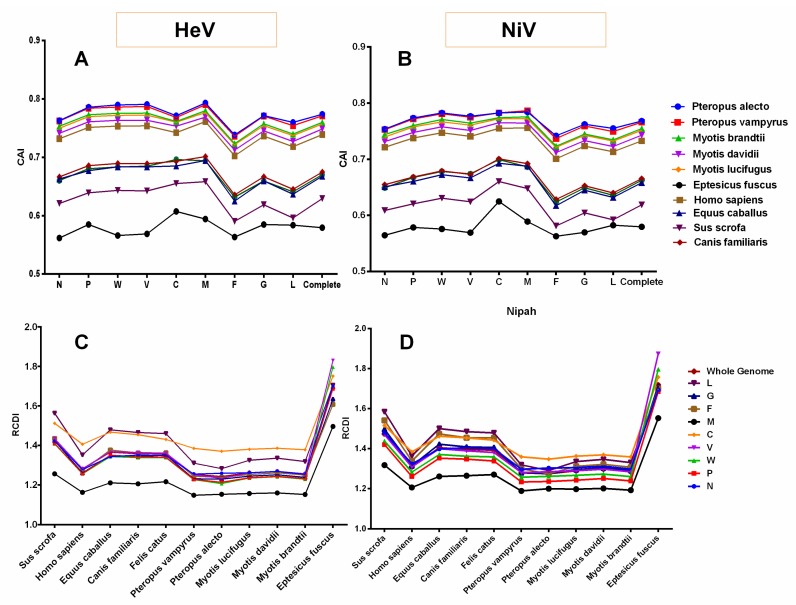
The Codon adaptation index (CAI) and relative codon deoptimization index (RCDI) analyses of HeV (**A**,**C**) and NiV (**B**,**D**) coding sequences in relation to different host species.

The codon usage similarities of the henipaviruses coding sequences with different hosts coding sequences were investigated through CAI analysis and a wide range of mean CAI values in the different hosts was observed (Figure 6A,B).

For instance, among the different *bat* species, the highest mean CAI values of the NiV coding sequences were observed for *Pteropus alecto* (0.767 ± 0.015), and the lowest for *Eptesicus fuscus* (0.579 ± 0.018) (Figure 6B). However, in mammals, the mean CAI values of the NiV coding sequences for *Homo sapiens* (0.732 ± 0.019) were considerably higher and the lowest in *Sus scrofa* (0.618 ± 0.025). In addition, M, C and W protein-coding genes had the highest mean CAI values while F had the lowest irrespective of the host species (Figure 6B and Supplementary Table S5). In the case of HeV, a similar trend in the species-wise CAI values as that of NiV was observed (Figure 6A). The M, V, W and P protein-coding genes in all the *bat* species and *Homo sapiens*, while M, C, W and V protein-coding genes in *Canis familiaris*, *Felis catus*, *Equus caballus* and *Sus scrofa* had the highest mean CAI values. Similar to NiV, F had the lowest CAI values for HeV irrespective of the host species (Figure 6A and Supplementary Table S5).

### 3.9. Henipaviruses Coding Sequences Showed Lowest Codon Usage Deoptimization for Pteropus alecto

The RCDI values of HeV and NiV coding sequences were computed to understand the codon usage deoptimization in relation to different host species. In both the cases of HeV and NiV, the mean RCDI for *Pteropus alecto* (RCDI_HeV_ = 1.243 ± 0.06, RCDI_Ni_V = 1.274 ± 0.04) was found to be the lowest while it was highest for *Eptesicus fuscus* (RCDI_HeV_ = 1.690 ± 0.10, RCDI_NiV_ = 1.718 ± 0.09). In addition, the mean RCDI for *Sus scrofa* was significantly higher as compared to all the bat species studied and *Homo sapiens* (*p* < 0.05–0.0001). Furthermore, the codon usage deoptimization in the individual protein-coding sequences of HeV and NiV was examined with respect to different host species. In general, the M protein-coding sequence had the lowest RCDI in all the host species. Of note, C protein-coding sequence showed the highest RCDI in *Homo sapiens* and *bat* species of this study (except *Eptesicus fuscus*) in both HeV and NiV (*p* < 0.05–0.0001), whilst in other species (*Equus caballus*, *Canis familiaris*, *Felis catus* and *Sus scrofa*), L had the highest RCDI values in general (Figure 6C,D).

### 3.10. Sus scrofa Had a High Similarity Index for Henipaviruses

**Figure 7:**
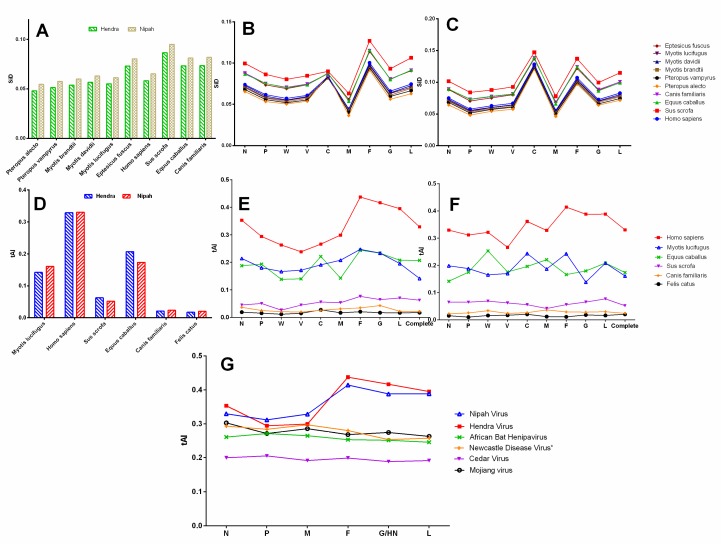
A comparative similarity index (SiD) analysis of complete genomic coding sequences of HeV and NiV (**A**), and individual protein-coding sequences of HeV (**B**) and NiV (**C**) are presented. The tRNA adaptation index (tAI) analysis of complete genomic coding sequences of HeV and NiV (**D**), individual protein-coding sequences of HeV (**E**) and NiV (**F**) are also presented. A comparison of tAI of HeV and NiV with another member of genus *Henipavirus*, Cedar virus (NCBI Reference Sequence: NC_025351) [2] and henipa-like viruses—African Bat Henipavirus (NCBI Reference Sequence: NC_025256) [42]; Mojiang virus (NCBI Reference Sequence: NC_025352) [43]; and distantly related Newcastle disease virus (NCBI Reference Sequence: NC_002617) is presented in (**G**).

A similarity index (SiD) analysis was carried out to inspect the influence of codon usage patterns of different host species on the evolution of the codon usage patterns of the HeV and NiV coding sequences. It was observed that *Sus scrofa* might have induced strong selection pressure (high SiD) on the coding sequences of HeV and NiV followed by *Equus caballus* and *Canis familiaris*, and minimum by *Pteropus alecto* (Figure 7A). Furthermore, to examine whether the influence of different host species on the individual protein-coding sequences of HeV and NiV follow a similar pattern, SiD values for each of the individual proteins coding sequences of HeV and NiV were calculated. In comparison to other coding sequences, F of HeV and C of NiV protein-coding sequences were more strongly influenced by all the host species, and M was the least influenced (Figure 7B,C). Overall, the influence of *Sus scrofa* was observed to be higher than *Equus caballus, Canis familiaris* and *Homo sapiens*. Besides, *Pteropus alecto* had the least influence on both the HeV and NiV coding sequences.

### 3.11. Henipaviruses Are Better Adapted to tRNAs Pool of Homo sapiens

The tAI of the coding sequences of HeV and NiV with respect to different host species was calculated and the influence of translational selection on the codon usage bias was assessed. In the case of NiV, a strong positive correlation between tAI and codon usage bias in all the species was observed, which implies that translation selection significantly influenced the codon usage bias in NiV coding sequences, irrespective of the host species. Furthermore, NiV showed a comparatively higher adaptation to the tRNAs pool of *Homo sapiens* (Tai = 0.330 ± 0.0019) followed by *Equus caballus* (tAI = 0.173 ± 0.0009), *Myotis lucifugus* (tAI = 0.160 ± 0.0009), *Sus scrofa* (tAI = 0.062 ± 0.0002), *Canis familiaris* (tAI = 0.023 ± 0.0002) and the lowest to *Felis catus* (tAI = 0.020 ± 0.00005) (Figure 7D). Considerably distinct translation efficiency patterns of different protein-coding sequences of NiV were observed in their host species. For instance, translation efficiencies of certain gene segments in *Homo sapiens* (F, G, L, C), *Myotis* (C, F), *Equus caballus* (W, M), and *Sus scrofa* (L) were found to be the highest (Figure 7F).

In the case of HeV, a strong positive correlation was observed for *Homo sapiens* (*r* = 0.84, *p* < 0.001) and *Sus scrofa* (*r* = 0.77, *p* < 0.01). Unlike NiV, translation selection influenced the codon usage bias in the coding sequences of HeV only in these two host species. Coincidentally, HeV showed a similar pattern of adaptation to tRNAs pool of host species as that of NiV (Figure 7E). In general, F and G of HeV showed a high translation efficiency in all the host species, while P-gene products had the lowest.

Furthermore, we calculated the tAI values of the recently identified henipa-like viruses (Mojiang virus, African bat henipavirus, and Cedar virus) for *human* cells and compared with that of henipaviruses (Figure 7G). Of note, both HeV and NiV showed a higher adaptability to the tRNAs pool of *Homo sapiens* as compared to henipa-like viruses, and that was predominantly due to higher adaptability of F, G, and L proteins (*p* < 0.05–0.001).

## 4. Discussion

The sequence data for henipaviruses available so far is limited and may pose misleading interpretations, especially in the evolutionary studies. To counteract the pitfalls associated with a small data set, we employed various state-of-the-art methods to explore the key factors responsible for the codon usage bias in henipaviruses and their evolution and adaptation to different hosts. In this study, the outcomes of these methods and corresponding interpretations have been summarized in a stepwise manner. The phylogenetic analysis suggested that HeV and NiV had easily differential evolutionary patterns. The NiV had evolved into two clades that separated Malaysian (genotype M) and Bangladeshi (genotype B) isolates. These clades had distinct temporal and geographical introductions viz. Clade I (genotype B) in 1995 and clade II (genotype M) in 1985 [44]. The results of the COA analysis performed on the RSCU of NiV isolates formed separate clusters based on the genotypes/clades, which were consistent with the phylogenetic analysis. Furthermore, a similar clustering was observed when the COA was performed at the individual gene level of NiV, indicating that these two clusters had independent geographical introductions (Figure 2). In the case of HeV, individual spillover events reported so far were likely to have occurred after exposure the HeV variants present in the fruit bat population in Australia [45]. Unlike NiV, there were no distinct clusters of HeV isolates, as revealed in the phylogenetic analysis. These insights were in concordance with the COA analysis of HeV isolates, where a random distribution of individual HeV isolate was observed on the two principal axes of the COA (Figure 3). However, at the individual gene level, the clustering overlapped among the different HeV isolates, indicating that HeV isolates experienced an evolutionary divergence from a common geographical introduction.

Codon bias is a common phenomenon across the genomes of several organisms and contributes significantly to the genome evolution. It is chiefly dictated via mutational pressure and natural selection; however, various other factors also contribute to the overall codon usage patterns. In this study, extensive analyzing methods were employed to comprehend these factors in the coding sequences of henipaviruses in a stepwise manner and we explored their role in the virus evolution and host adaptation. Here, we first analyzed the nucleotide compositions in the coding sequences of henipaviruses and correlated it with their RSCU patterns. The henipavirsues were rich in AU as compared to GC content and all the over- and under-represented codons in them were A/U or G/C-ended, respectively. The dinucleotide CpG had been the most frequently seen in these under-represented codons. During the course of evolution, the viruses tended to reduce their CpG compositions to avoid the strong stimulation of innate immune response by Toll-like receptor 9 (TLR9), a type of intracellular pattern recognition receptor (for unmethylated CpG) [19]. Thus, CpG deficiency contributed significantly in shaping the codon usage patterns in henipaviruses. Since the viruses depend on their hosts for their replication, selection of optimal codons are largely influenced by their host, especially due to translation selection. It is to note that the dinucleotide composition in RNA viruses is a true representation of its virus family and reflects poorly on its host species [46]. On comparing the RSCU patterns of henipaviruses with their hosts, we came across similar findings where the henipaviruses showed a poor correlation of RSCU pattern (in terms of usage of most preferred codons) with that of their hosts. Such types of observations have been reported previously in poliovirus and hepatitis A virus [39,47]. This poorly correlated RSCU pattern though is expected to reduce the translation efficiency, but at the same time, might allow the virus proteins to fold properly [48].

Furthermore, the ENc values were calculated in the complete coding sequences of henipaviruses and also in their individual protein-coding genes to estimate the overall codon usage bias. The overall codon usage bias was found to be slightly lower among the coding sequences of henipaviruses (HeV_ENc_ = 51.21 ± 0.07, NiV_ENc_ = 51.06 ± 0.38). A similar kind of slightly low codon usage bias has also been reported among several other RNA viruses, such as Zika virus (ENc = 53.32) [49], Ebola virus (ENc = 57.23) [50], Equine Influenza virus (ENc = 52.09) [51], and Hepatitis C virus (ENc = 52.62) [48]. Apart from this, the presence of variations on the codon bias exhibited by the different virus segments could be attributed to different selection pressures acting over the respective proteins. A slightly low codon bias in the RNA viruses is proposed to have a selective advantage for their efficient replication as it might reduce the competition for synthesis machinery between the viruses and their hosts [51]. Since the henipaviruses and their hosts differed significantly in RSCUs or usage of ‘most preferred codon for a particular amino acid’, the evolution of these viruses to have a slightly low codon usage bias might have favored their maintenance and replication in the different host species.

The ENc values provide an overview of overall codon usage bias; however, the primary factors (directional mutation pressure and/or natural selection) responsible for this codon bias could not be deduced from these values. To elucidate the magnitude of the underlying factors of the codon bias observed in the complete coding sequences of henipaviruses, PR-2, ENc–GC3s, and neutrality plots were generated. The natural selection dominantly influenced the overall codon usage patterns in the henipaviruses (90.24% in HeV and 94.47% in NiV). However, codon usage patterns in the coding sequence of C protein of NiV were predominantly influenced by the directional mutation pressure (57.3%). A significant influence of mutation pressure on the codon usage patterns in W (33.28%) and V (42.15%) proteins of HeV was also observed (Supplementary Table S4).

It has been postulated that high CAI values are associated with a predominance of natural selection in shaping the codon usage patterns [52]. Therefore, the influence of natural selection was further confirmed through CAI analysis, which also demonstrated the adaptation of viral genes to their hosts. On the basis of CAI analysis of the complete coding sequences of henipaviruses, various levels of adaptation to different host species were observed. Typically, the highest level of adaptation of henipaviruses (both HeV and NiV) was in an established reservoir host of the genus *Pteropus* (megabats–*P. alecto*, *P. vampyrus*) followed by microbats (*M. brandtii*, *M. lucifugus*, *M. davidii*), the clinical hosts (*Homo sapiens*, *Canis familiaris*, *Felis catus*) and intermediate hosts (*Equus caballus*, *Sus scrofa*). A comparatively lower CAI in the intermediate hosts could be inferable from either low efficiency of replication or transient protein expression in these hosts.

Two additional codon usage indices, RCDI and SiD, were also performed to further evaluate the adaptation of henipaviruses to different host species. As per the RCDI analysis, henipaviruses coding sequences had the lowest RCDI values for *P. alecto* and the highest for the *S. scrofa*. A low RCDI value might indicate the strong adaptation to a host and vice-versa, which is consistent with the high CAI values of the henipaviruses for *P. alecto*. Conversely, a high RCDI value indicates that the virus is less adaptive to its host, which may be attributable to low replication or the expression of some of the virus genes in the latency phases. However, such a virus could employ an alternative codon usage pattern for its successful establishment in a host [38]. Furthermore, SiD analysis revealed that *P. alecto* might have induced the least selection pressure on the coding sequences on the henipaviruses while *S. scrofa* might have induced the highest. The CAI, RCDI and SiD analyses suggested that henipaviruses are well adapted to a reservoir host of genus *Pteropus*, resulting in minimal pressure to change over a period of time. Additionally, henipaviruses also showed a high level of adaptation to microbats of the genus *Myotis*. Our findings could be connected to a growing evidence that the henipaviruses may infect other genera of *pteropodid bats* (*fruit bats* in the family *Pteropodidae*), as well as *microbats*. For instance, serological evidence of circulation of henipaviruses, or henipa-like viruses in microbats (*Myotis* spp. in China and *Scotophilus kuhli* in Malaysia) has been reported [14,53]. Interestingly, the analyses suggested that henipaviruses might not be well adapted to another *microbat*, *E. fuscus*. Whether *microbats* could sustain or maintain henipavirus infection as that of observed in *Pteropus* needs further experimental investigations. 

Two separate clusters of NiV, Malaysian (genotype M) and Bangladeshi (genotype B), as evident from the phylogenetic and COA analyses, have quite distinct transmission cycles. For instance, the major pathway of NiV transmission in Malaysia has been *bat*-*pig*, *pig*-*pig*, and *pig*-*human*, while in Bangladesh, *bat*-*human* and *human*-*human* transmission is frequent without the involvement of an intermediate host (like *horse*, *pig*, *cat*, *dog*) [54,55]. The key differentiating features of Bangladesh NiV isolates have been the *human-human* transmission, predominant respiratory involvement, and high *human* fatality rates, which form a clear epidemiological boundary that separates it from Malaysian isolates. The underlying risk factors for these characteristic Bangladesh NiV infections have been mapped to drinking date palm sap contaminated with *bat* urine/saliva, and close contact with a patient with NiV encephalitis [56]. Furthermore, the poor care and medical practices in Bangladesh/India compared to its Malaysian counterpart cannot be neglected for these high case fatalities. Besides, the Bangladesh NiV isolate replicated more efficiently in the *human* tracheal/bronchial cells as compared to the Malaysian NiV isolate [57]. We speculate that the high replication efficiency of the Bangladesh NiV might be associated with (i) a comparatively high translation efficiency of G and W proteins of Bangladesh NiV isolates in *humans* (as evident from a slightly high tAI of Bangladesh NiV isolates (G = 0.395 ± 0.001; W = 0.324 ± 0.001) as compared to the Malaysian isolates (G = 0.384 ± 0003; W = 0.319 ± 0.0002)) (Supplementary Table S6), and (ii) a high relative CpG frequency in the immune-modulating genes (P, W and V) of the Malaysian isolates that might restrict their efficient replication as compared to the Bangladesh isolates (Supplementary Figure S2).

Next, the tRNA adaptation index (tAI), an indicative of translation efficiency, was estimated using a new approach which calculates the tAI based on the species-specific tAI wobble weights [35]. The results showed that both HeV and NiV are well adapted to tRNAs pool of *humans*, but are poorly adapted to *pig*’s tRNAs pool. This finding corroborated with an earlier study where NiV showed increased replication in *humans* as compared to *pig* primary airway epithelial cell cultures [58]. Furthermore, the gene-wise translation efficiency of henipaviruses was also calculated. In the case of HeV, a common trend in the translation efficiency of different gene segments in all the hosts viz. high (F and G) and low (W, V and C) translation efficiencies except *Equus caballus* (C showed a comparable high translation efficiency in *Equus caballus* which might allow them to counteract the host antiviral system efficiently) were observed. The gene segments of NiV also showed a diverse pattern of translation efficiencies in their hosts viz. translation efficiency in *Homo sapiens* (F, G, L, C), *Myotis* (C, F), *Equus caballus* (W, M), and *Sus scrofa* (L) were the highest. This study provided the evidence that among the different proteins encoded by the NiV in *Myotis*, the translation efficiency of C protein was the highest (however, the translation efficiency of C protein in *Pteropus* could not be estimated due to non-availability of *Pteropus* tRNA copies in the GtRNAdb: Genomic tRNA Database). The kinetics of P-gene products of NiV in *human* cells showed the abundance of P transcripts at the initial stage of infection followed by V and W transcripts as the infection progressed to counteract the interferon (IFN)-induced antiviral responses of their hosts [59]. It is worth noting that henipaviruses P-gene product’s kinetics or functions have been studied in *human* cells only. The kinetics of P-gene products in the chiropteran cells has not been studied yet and it would be of interest to determine experimentally the kinetics and key roles of P-gene products in the chiropteran cells. However, we speculate that high translation efficiency of the C of NiV into chiropteran cells might allow the NiV to evade the host innate immune system by decreasing the viral RNA synthesis and thereby help in setting up a subclinical infection. Recently, henipa-like viruses have been identified in *rat* (Mojiang virus) and *Eidolon helvum* (African bat henipavirus), which in our study, showed poor adaptation to *human*’s tRNAs pool, especially because of poor adaptation of their F, G and L proteins and thus, these viruses might pose a narrow risk of zoonotic transmission. These results are further supported by an earlier study which highlighted that African bat henipavirus possessed reduced biological activity in F and G proteins in most of the mammalian cells [42].

Given the evidence of (i) infection with henipaviruses in multiple *bat* species globally, (ii) increased spillover events of henipaviruses into *horse*/*pig*/*human* due to fast urbanization, (iii) higher adaptation to the tRNAs pool of *humans*; there exists a high probability of emergence of these viruses in the future, especially in *humans* after a close contact with *bat* excretions. It is, therefore, an active and extensive surveillance, especially in the *bat* population; an awareness of maintaining the hygienic conditions near the interface with forest/wildlife; and last but not least, avoiding direct/indirect contact with *bat* excretions appears to be the major preventative strategy against henipaviruses.

## Figures and Tables

**Table 1:** The Relative synonymous codon usage (RSCU) patterns of HeV and NiV with their host species.

Amino Acid	Codons	HeV	NiV	Bat *	*Human*	*Horse*	*Pig*	*Dog*	Amino Acid	Codons	HeV	NiV	Bat *	*Human*	*Horse*	*Pig*	*Dog*
Phe	UUU	1.08	1.14	0.95	0.93	0.83	0.79	0.82	Ser	UCA	**1.78**	**1.79**	1.02	0.90	0.80	0.73	0.81
	UUC	0.92	0.86	1.05	1.07	1.17	1.21	1.16		UCG	*0.30*	*0.27*	*0.37*	*0.33*	*0.34*	*0.39*	*0.38*
Leu	UUA	0.95	0.96	0.78	*0.46*	*0.33*	*0.32*	*0.35*		AGU	1.23	1.18	0.82	0.90	0.86	0.77	0.89
	UUG	1.05	1.04	1.22	0.77	0.72	0.67	0.68		AGC	0.77	0.83	1.18	1.44	1.48	**1.62**	1.56
	CUU	1.14	1.16	0.71	0.79	0.73	0.65	0.67	Arg	AGA	1.36	1.38	1.03	1.29	1.30	1.12	1.20
	CUC	0.94	0.78	0.96	1.17	1.32	1.35	1.25		AGG	0.64	0.62	0.97	1.27	1.32	1.23	1.32
	CUA	0.99	1.06	*0.39*	*0.43*	*0.34*	*0.33*	*0.37*		CGU	1.06	1.42	*0.59*	*0.48*	*0.55*	*0.44*	*0.46*
	CUG	0.94	1.01	**1.93**	**2.37**	**2.56**	**2.68**	**2.45**		CGC	0.76	*0.25*	1.18	1.10	1.15	1.31	1.26
Ile	AUU	1.02	1.03	1.11	1.08	0.92	0.91	0.96		CGA	**1.79**	**1.82**	0.82	0.65	0.61	0.60	0.67
	AUC	1.00	0.94	1.34	1.41	**1.66**	**1.67**	**1.61**		CGG	*0.39*	*0.52*	1.40	1.21	1.08	1.29	1.31
	AUA	0.99	1.03	*0.55*	*0.51*	*0.42*	*0.42*	*0.45*	Cys	UGU	1.23	1.31	0.96	0.91	0.89	0.79	0.85
Val	GUU	1.14	1.47	0.75	0.73	0.60	*0.57*	*0.58*		UGC	0.77	0.69	1.04	1.09	1.11	1.21	1.10
	GUC	1.01	0.75	0.95	0.95	1.08	1.07	1.10	His	CAU	1.43	1.19	0.86	0.84	0.81	0.70	0.78
	GUA	0.78	1.03	*0.52*	*0.47*	*0.35*	*0.34*	*0.42*		CAC	*0.57*	0.81	1.14	1.16	1.19	1.30	1.22
	GUG	1.07	0.75	**1.77**	**1.85**	**1.97**	**2.03**	**1.98**	Gln	CAA	1.17	1.26	*0.55*	*0.53*	*0.52*	*0.44*	*0.50*
Pro	CCU	1.44	**1.68**	1.18	1.15	1.19	1.05	1.08		CAG	0.83	0.74	1.46	1.47	1.48	1.56	1.46
	CCC	0.67	0.68	1.24	1.29	1.38	1.46	1.47	Asn	AAU	1.34	1.29	0.98	0.94	0.84	0.79	0.87
	CCA	1.42	1.16	1.13	1.11	0.97	0.94	1.05		AAC	0.66	0.71	1.02	1.06	1.16	1.21	1.12
	CCG	*0.47*	*0.48*	*0.45*	*0.45*	*0.45*	*0.56*	*0.51*	Lys	AAA	1.04	1.17	0.91	0.87	0.79	0.76	0.79
Thr	ACU	1.32	1.42	1.04	0.99	0.94	0.83	0.89		AAG	0.96	0.83	1.09	1.13	1.21	1.24	1.13
	ACC	0.69	0.71	1.33	1.42	1.58	**1.68**	1.58	Asp	GAU	1.34	1.25	0.95	0.93	0.83	0.80	0.86
	ACA	**1.71**	**1.66**	1.16	1.14	0.96	0.92	1.05		GAC	0.66	0.75	1.05	1.07	1.17	1.20	1.09
	ACG	*0.28*	*0.22*	*0.48*	*0.46*	*0.52*	*0.57*	*0.53*	Glu	GAA	1.12	1.12	0.90	0.84	0.76	0.72	0.79
Ala	GCU	1.53	1.51	1.09	1.06	1.05	0.96	1.00		GAG	0.88	0.89	1.10	1.16	1.24	1.28	1.23
	GCC	*0.57*	0.63	1.57	1.60	**1.72**	**1.80**	**1.78**	Gly	GGU	1.05	0.92	0.67	0.65	0.65	*0.57*	0.65
	GCA	**1.65**	1.59	0.94	0.91	0.77	0.74	0.81		GGC	*0.49*	*0.53*	1.31	1.35	1.43	1.46	1.45
	GCG	*0.25*	*0.28*	*0.40*	*0.42*	*0.45*	*0.50*	*0.47*		GGA	1.36	1.51	1.04	1.00	0.95	0.91	1.02
Tyr	UAU	1.14	1.14	0.92	0.89	0.75	0.73	0.79		GGG	1.10	1.05	0.98	1.00	0.97	1.05	1.05
	UAC	0.86	0.86	1.08	1.11	1.25	1.27	1.15	Trp	TGG	1.00	1.00	1.00	1.00	1.00	1.00	1.00
Ser	UCU	1.29	1.37	1.27	1.13	1.09	0.99	1.09	Met	ATG	1.00	1.00	1.00	1.00	1.00	1.00	1.00
	UCC	0.63	*0.56*	1.34	1.31	1.43	1.50	1.52									

Note: Over- (RSCU ≥ 1.6) and under-represented (RSCU ≤ 0.6) codons are displayed in bold and italics, respectively. * denotes *Pteropus alecto*.

## Supplementary Materials

The following are available online at http://www.mdpi.com/1999-4915/10/11/604/s1, Figure S1: PR-2 plots for the entire coding sequences of HeV and NiV isolates, Figure S2: A comparison of relative CpG frequencies of Malaysia and Bangladesh NiV isolates, Table S1: The demographics of HeV and NiV isolates used in this study, Table S2: The nucleotide compositions analyses of HeV and NiV isolates used in this study, Table S3: Correlation analyses among the nucleotide compositions and other parameters of NiV and HeV coding sequences, Table S4: Neutrality plots for entire coding sequences of NiV and HeV isolates, Table S5: The CAI values of different protein coding sequences of HeV and NiV in relation to various hosts, Table S6: The tRNA adaptation index for NiV isolates.
