# Supplementary material for: Evolution of Codon Usage Bias in Henipaviruses Is Governed by Natural Selection and Is Host-Specific
Source: Viruses. 2018 Nov 1;10(11):604. doi: 10.3390/v10110604 (PMC6266499; doi:10.3390/v10110604)
Supplement: Supplementary file 1 [file viruses-10-00604-s001.zip › viruses-371287-supplementary/Supplementary Data/Supplementary Figures captions.docx]

Figure S1: PR-2 plots for the entire coding sequences of HeV and NiV isolates.

Figure S2: A comparison of relative CpG frequencies of Malaysia and Bangladesh NiV isolates
