# Supplementary material for: Evolution of Codon Usage Bias in Henipaviruses Is Governed by Natural Selection and Is Host-Specific
Source: Viruses. 2018 Nov 1;10(11):604. doi: 10.3390/v10110604 (PMC6266499; doi:10.3390/v10110604)
Supplement: Supplementary file 1 [file viruses-10-00604-s001.zip › viruses-371287-supplementary/Supplementary Data/Supplementary Table 1.doc]

**Supplementary Table 1: The demographics of HeV and NiV isolates used in this study.**

| **S.Nos.** | **Virus Isolates** | **GenBank Acces. No.** | **Genome Length** | **Collection Date** | **Host** | **Country** |
| --- | --- | --- | --- | --- | --- | --- |
|  | **HeV isolates** | | | | | |
| 1 | HeV/Australia/Bat/2009/CedarGrove 11c | JN255803 | 18234 | 8/5/2009 | Bat | Australia |
| 2 | HeV/Australia/Bat/2009/TolgaScrub 30g | JN255802 | 18234 | 8/24/2009 | Bat | Australia |
| 3 | HeV/Australia/Bat/2009/Yeppoon Y47a | JN255800 | 18234 | 8/15/2009 | Bat | Australia |
| 4 | HeV/Australia/Bat/2009/Yeppoon Y4a | JN255801 | 18234 | 8/13/2009 | Bat | Australia |
| 5 | HeV/Australia/Horse/2006/Murwillumbah | HM044318 | 18234 | 10/1/2006 | Horse | Australia |
| 6 | HeV/Australia/Horse/2007/Clifton Beach Q | JN255804 | 18234 | 7/1/2007 | Horse | Australia |
| 7 | HeV/Australia/Horse/2007/CliftonBeach | HM044321 | 18234 | 7/1/2007 | Horse | Australia |
| 8 | HeV/Australia/Horse/2007/Peachester | HM044319 | 18234 | 6/1/2007 | Horse | Australia |
| 9 | HeV/Australia/Horse/2008/Proserpine | HM044320 | 18234 | 7/1/2008 | Horse | Australia |
| 10 | HeV/Australia/Horse/2008/Redlands | HM044317 | 18234 | 7/1/2008 | Horse | Australia |
| 11 | HeV/Australia/Horse/2009/Cawarral | JN255806 | 18234 | 8/1/2009 | Horse | Australia |
| 12 | HeV/Australia/Human Female/2008/Redlands | JN255805 | 18234 | 7/1/2008 | Human | Australia |
| 13 | HeV/Australia/Horse/1994 | AF017149 | 18234 | 1994 | Horse | Australia |
|  | **NiV isolates** | | | | | |
| 1 | NiV/ Malaysia/Bat/2008 | FN869553 | 14973 | 2008 | Bat | Malaysia |
| 2 | NiV/Malaysia/Pig/1999/99/VRI-0626 | AJ627196 | 18246 | 1999 | Pig | Malaysia |
| 3 | NiV/Malaysia/Pig/1999/99/VRI-2794 | AJ564621 | 18246 | 1999 | Pig | Malaysia |
| 4 | NiV/Malaysia/Pig/1999/99/VRI-1413 | AJ564622 | 18246 | 1999 | Pig | Malaysia |
| 5 | NiV/Malaysia/Pig/1999/99/UM-0128 | AJ564623 | 18246 | 1999 | Pig | Malaysia |
| 6 | NiV/Malaysia/Human/1999/ UMMC1 | AY029767 | 18246 | 1999 | Human | Malaysia |
| 7 | NiV/Malaysia/Human/1999/ UMMC2 | AY029768 | 18246 | 1999 | Human | Malaysia |
| 8 | NiV/Malaysia/Human/1999 | AF212302 | 18246 | 1999 | Human | Malaysia |
| 9 | NiV/Bangladesh/Human/2008/NIVBGD2008MANIKGONJ | JN808857 | 18252 | 2008 | Human | Bangladesh |
| 10 | NiV/Bangladesh/Human/2008/ NIVBGD2008RAJBARI | JN808863 | 18252 | 2008 | Human | Bangladesh |
| 11 | NiV/Bangladesh/Human/2008/ NIVBGD2010FARIDPUR | JN808864 | 18252 | 2010 | Human | Bangladesh |
| 12 | NiV/India/Human/2007/ Ind-Nipah-07-FG | FJ513078 | 18252 | 2007 | Human | India |
| 13 | NiV/Bangladesh/Human/2004 | AY988601 | 18252 | 2004 | Human | Bangladesh |
