# Supplementary material for: Evolution of Codon Usage Bias in Henipaviruses Is Governed by Natural Selection and Is Host-Specific
Source: Viruses. 2018 Nov 1;10(11):604. doi: 10.3390/v10110604 (PMC6266499; doi:10.3390/v10110604)
Supplement: Supplementary file 1 [file viruses-10-00604-s001.zip › viruses-371287-supplementary/Supplementary Data/Supplementary Table 2.doc]

**Supplementary Table 2A: The nucleotide compositions analyses of HeV isolates used in this study.**

| **HeV Isolates** | **A (%)** | **C (%)** | **G (%)** | **U (%)** | **U3(%)** | **C3(%)** | **A3(%)** | **G3(%)** | **AU(%)** | **GC(%)** | **GC1** | **GC2** | **GC3** | **GC12** | **ENC** | **GC3s** |
| --- | --- | --- | --- | --- | --- | --- | --- | --- | --- | --- | --- | --- | --- | --- | --- | --- |
| JN255806 | 32.59 | 18.93 | 21.9 | 26.58 | 38.32 | 23.74 | 39.96 | 25.39 | 59.17 | 40.83 | 45.78 | 37.26 | 39.43 | 41.52 | 51.27 | 0.369 |
| JN255805 | 32.64 | 18.9 | 21.84 | 26.61 | 38.47 | 23.66 | 40.07 | 25.25 | 59.25 | 40.74 | 45.80 | 37.16 | 39.24 | 41.48 | 51.13 | 0.365 |
| JN255804 | 32.63 | 18.88 | 21.84 | 26.65 | 38.52 | 23.66 | 40.01 | 25.24 | 59.28 | 40.72 | 45.72 | 37.16 | 39.26 | 41.44 | 51.2 | 0.365 |
| JN255803 | 32.6 | 18.88 | 21.87 | 26.64 | 38.49 | 23.61 | 39.97 | 25.37 | 59.24 | 40.75 | 45.76 | 37.18 | 39.31 | 41.47 | 51.21 | 0.368 |
| JN255802 | 32.62 | 18.87 | 21.87 | 26.64 | 38.49 | 23.59 | 40.02 | 25.34 | 59.26 | 40.74 | 45.76 | 37.18 | 39.26 | 41.47 | 51.18 | 0.367 |
| JN255801 | 32.62 | 18.87 | 21.87 | 26.64 | 38.49 | 23.59 | 40.02 | 25.34 | 59.26 | 40.74 | 45.76 | 37.18 | 39.26 | 41.47 | 51.18 | 0.367 |
| JN255800 | 32.62 | 18.87 | 21.87 | 26.64 | 38.49 | 23.59 | 40.02 | 25.34 | 59.26 | 40.74 | 45.76 | 37.18 | 39.26 | 41.47 | 51.18 | 0.367 |
| HM044321 | 32.62 | 18.91 | 21.86 | 26.61 | 38.4 | 23.75 | 39.99 | 25.3 | 59.23 | 40.77 | 45.76 | 37.14 | 39.39 | 41.45 | 51.27 | 0.368 |
| HM044320 | 32.62 | 18.89 | 21.87 | 26.61 | 38.44 | 23.64 | 40.05 | 25.28 | 59.23 | 40.76 | 45.80 | 37.20 | 39.26 | 41.50 | 51.23 | 0.366 |
| HM044319 | 32.59 | 18.91 | 21.91 | 26.58 | 38.41 | 23.63 | 39.94 | 25.46 | 59.17 | 40.82 | 45.84 | 37.24 | 39.39 | 41.54 | 51.29 | 0.371 |
| HM044318 | 32.65 | 18.97 | 21.82 | 26.55 | 38.26 | 23.92 | 40.01 | 25.24 | 59.2 | 40.79 | 45.74 | 37.16 | 39.47 | 41.45 | 51.13 | 0.366 |
| HM044317 | 32.64 | 18.9 | 21.84 | 26.61 | 38.47 | 23.66 | 40.07 | 25.25 | 59.25 | 40.74 | 45.80 | 37.16 | 39.24 | 41.48 | 51.13 | 0.365 |
| AF017149 | 32.53 | 19.0 | 21.91 | 26.56 | 38.22 | 23.96 | 39.75 | 25.48 | 59.12 | 40.90 | 45.80 | 37.20 | 39.72 | 41.50 | 51.36 | 0.342 |
| **Mean** | **32.61** | **18.91** | **21.87** | **26.61** | **38.42** | **23.69** | **39.99** | **25.33** | **59.22** | **40.77** | **45.78** | **37.19** | **39.35** | **41.48** | **51.21** | **0.365** |
| **SD** | **0.03** | **0.038** | **0.027** | **0.032** | **0.092** | **0.117** | **0.079** | **0.077** | **0.034** | **0.033** | **0.030** | **0.032** | **0.124** | **0.028** | **0.067** | **0.007** |

**Supplementary Table 2B: The nucleotide compositions analyses of NiV** isolates used in this study.

| **NiV Isolates** | **A(%)** | **C(%)** | **G(%)** | **U(%)** | **U3(%)** | **C3(%)** | **A3(%)** | **G3(%)** | **AU(%)** | **GC(%)** | **GC1** | **GC2** | **GC3** | **GC12** | **ENC** | **GC3s** |
| --- | --- | --- | --- | --- | --- | --- | --- | --- | --- | --- | --- | --- | --- | --- | --- | --- |
| NC_002728.1 | 33.22 | 18.73 | 21.19 | 26.86 | 39.32 | 23.18 | 41.59 | 23.43 | 60.08 | 39.92 | 45.39 | 36.88 | 37.48 | 41.14 | 50.76 | 0.30 |
| FJ513078 | 32.99 | 18.89 | 21.31 | 26.8 | 39.08 | 24.04 | 40.51 | 24.05 | 59.79 | 40.2 | 45.37 | 36.72 | 38.53 | 41.04 | 51.59 | 0.31 |
| AF212302 | 33.22 | 18.73 | 21.19 | 26.86 | 39.32 | 23.18 | 41.59 | 23.43 | 60.08 | 39.92 | 45.39 | 36.88 | 37.48 | 41.14 | 50.76 | 0.30 |
| JN808863 | 32.98 | 18.86 | 21.36 | 26.8 | 39.16 | 23.89 | 40.48 | 24.22 | 59.78 | 40.22 | 45.41 | 36.72 | 38.53 | 41.06 | 51.61 | 0.31 |
| JN808864 | 33.01 | 18.93 | 21.33 | 26.73 | 38.9 | 24.13 | 40.64 | 24.02 | 59.74 | 40.26 | 45.43 | 36.72 | 38.61 | 41.08 | 51.52 | 0.31 |
| JN808857 | 32.98 | 18.86 | 21.36 | 26.8 | 39.16 | 23.89 | 40.48 | 24.22 | 59.78 | 40.22 | 45.41 | 36.72 | 38.53 | 41.06 | 51.61 | 0.31 |
| FN869553 | 33.19 | 18.84 | 21.25 | 26.72 | 38.99 | 23.47 | 41.52 | 23.54 | 59.91 | 40.09 | 45.51 | 36.94 | 37.81 | 41.23 | 50.91 | 0.30 |
| AY029768 | 33.22 | 18.73 | 21.19 | 26.87 | 39.34 | 23.16 | 41.59 | 23.43 | 60.09 | 39.92 | 45.39 | 36.88 | 37.46 | 41.14 | 50.75 | 0.30 |
| AY029767 | 33.22 | 18.73 | 21.19 | 26.87 | 39.32 | 23.18 | 41.6 | 23.43 | 60.09 | 39.92 | 45.39 | 36.86 | 37.48 | 41.13 | 50.76 | 0.30 |
| AY988601 | 32.99 | 18.91 | 21.33 | 26.77 | 38.98 | 24.07 | 40.67 | 23.93 | 59.76 | 40.24 | 45.43 | 36.78 | 38.51 | 41.11 | 51.49 | 0.31 |
| AJ627196 | 33.23 | 18.71 | 21.2 | 26.87 | 39.27 | 23.17 | 41.65 | 23.47 | 60.1 | 39.91 | 45.43 | 36.82 | 37.46 | 41.13 | 50.78 | 0.30 |
| AJ564623 | 33.23 | 18.72 | 21.18 | 26.88 | 39.32 | 23.18 | 41.62 | 23.4 | 60.11 | 39.9 | 45.37 | 36.86 | 37.46 | 41.12 | 50.76 | 0.30 |
| AJ564622 | 33.22 | 18.73 | 21.19 | 26.87 | 39.32 | 23.18 | 41.59 | 23.43 | 60.09 | 39.92 | 45.37 | 36.88 | 37.48 | 41.13 | 50.76 | 0.30 |
| AJ564621 | 33.23 | 18.72 | 21.18 | 26.88 | 39.34 | 23.16 | 41.62 | 23.4 | 60.11 | 39.9 | 45.39 | 36.86 | 37.44 | 41.13 | 50.75 | 0.30 |
| **Mean** | **33.13** | **18.79** | **21.24** | **26.82** | **39.20** | **23.49** | **41.22** | **23.67** | **59.96** | **40.03** | **45.40** | **36.82** | **37.87** | **41.11** | **51.05** | **0.30** |
| **SD** | **0.110** | **0.080** | **0.071** | **0.054** | **0.150** | **0.393** | **0.501** | **0.319** | **0.153** | **0.148** | **0.035** | **0.0734** | **0.501** | **0.042** | **0.38** | **0.003** |
