# Supplementary material for: Evolution of Codon Usage Bias in Henipaviruses Is Governed by Natural Selection and Is Host-Specific
Source: Viruses. 2018 Nov 1;10(11):604. doi: 10.3390/v10110604 (PMC6266499; doi:10.3390/v10110604)
Supplement: Supplementary file 1 [file viruses-10-00604-s001.zip › viruses-371287-supplementary/Supplementary Data/Supplementary Table 3.doc]

**Supplementary Table 3A: Correlation analyses among the nucleotide compositions and other parameters of NiV coding sequences.**

| **Variables** | | **A(%)** | | **C(%)** | | **G(%)** | | **U(%)** | | **U3(%)** | | **C3(%)** | | **A3(%)** | | **G3(%)** | | **AU(%)** | | **GC(%)** | | **GC3** | | **GC12** | | **ENC** | | **GC3s** | | **CAI** | |
| --- | --- | --- | --- | --- | --- | --- | --- | --- | --- | --- | --- | --- | --- | --- | --- | --- | --- | --- | --- | --- | --- | --- | --- | --- | --- | --- | --- | --- | --- | --- | --- |
| **A(%)** |  | |  | |  | |  | |  | |  | |  | |  | |  | |  | |  | |  | |  | |  | |  | |  |
| **C(%)** | **-0.912***** | |  | |  | |  | |  | |  | |  | |  | |  | |  | |  | |  | |  | |  | |  | |  |
| **G(%)** | **-0.893***** | | **0.800**** | |  | |  | |  | |  | |  | |  | |  | |  | |  | |  | |  | |  | |  | |  |
| **U(%)** | **0.818**** | | **-0.869***** | | **-0.822**** | |  | |  | |  | |  | |  | |  | |  | |  | |  | |  | |  | |  | |  |
| **U3(%)** | **0.701*** | | **-0.786**** | | **-0.827**** | | **0.876***** | |  | |  | |  | |  | |  | |  | |  | |  | |  | |  | |  | |  |
| **C3(%)** | **-0.828**** | | **0.913***** | | **0.787**** | | **-0.835**** | | **-0.903***** | |  | |  | |  | |  | |  | |  | |  | |  | |  | |  | |  |
| **A3(%)** | **0.977***** | | **-0.906***** | | **-0.846**** | | **0.805**** | | **0.660*** | | **-0.811**** | |  | |  | |  | |  | |  | |  | |  | |  | |  | |  |
| **G3(%)** | **-0.896***** | | **0.788**** | | **0.985***** | | **-0.800**** | | **-0.805**** | | **0.775*** | | **-0.861***** | |  | |  | |  | |  | |  | |  | |  | |  | |  |
| **AU(%)** | **0.915***** | | **-0.956***** | | **-0.877***** | | **0.922***** | | **0.819**** | | **-0.898***** | | **0.902***** | | **-0.847**** | |  | |  | |  | |  | |  | |  | |  | |  |
| **GC(%)** | **-0.930***** | | **0.972***** | | **0.892***** | | **-0.897***** | | **-0.816**** | | **0.893***** | | **-0.906***** | | **0.861***** | | **-0.983***** | |  | |  | |  | |  | |  | |  | |  |
| **GC3** | **-0.917***** | | **0.931***** | | **0.868***** | | **-0.865***** | | **-0.834**** | | **0.940***** | | **-0.913***** | | **0.876***** | | **-0.940***** | | **0.940***** | |  | |  | |  | |  | |  | |  |
| **GC12** | **0.557*** | | -0.519 | | **-0.577**** | | 0.223 | | 0.487 | | **-0.631*** | | **0.544*** | | **-0.611*** | | 0.454 | | -0.494 | | **-0.588*** | |  | |  | |  | |  | |  |
| **ENC** | **-0.839**** | | **0.738*** | | **0.931***** | | **-0.796**** | | **-0.842**** | | **0.829**** | | **-0.817**** | | **0.946***** | | **-0.830**** | | **0.802**** | | **0.883***** | | **-0.629*** | |  | |  | |  | |  |
| **GC3s** | **-0.829**** | | **0.837**** | | **0.945***** | | **-0.876***** | | **-0.873***** | | **0.814**** | | **-0.790**** | | **0.909***** | | **-0.916***** | | **0.909***** | | **0.831**** | | -0.486 | | **0.863***** | |  | |  | |  |
| **CAI** | -0.315 | | 0.013 | | 0.407 | | -0.292 | | -0.152 | | 0.006 | | -0.299 | | 0.462 | | -0.135 | | 0.138 | | 0.236 | | 0.124 | | 0.411 | | 0.208 | |  | |  |
| **Axis1** | **0.786**** | | **-0.692*** | | **-0.885***** | | **0.676*** | | **0.788**** | | **-0.758*** | | **0.748*** | | **-0.900***** | | **0.718*** | | **-0.741*** | | **-0.775*** | | **0.698*** | | **-0.853**** | | **-0.831**** | | -0.363 | |  |
| **Axis2** | 0.263 | | -0.014 | | -0.133 | | 0.087 | | 0.029 | | -0.070 | | 0.275 | | -0.221 | | -0.029 | | 0.018 | | -0.175 | | 0.152 | | -0.272 | | 0.115 | | **-0.591*** | |  |

Note: *****p<0.0001; **p<0.001; *p<0.05**

**Supplementary Table 3B: Correlation analyses among the nucleotide compositions and other parameters of** HeV coding sequences.

| **Variables** | **A (%)** | **C (%)** | **G (%)** | **U (%)** | **U3(%)** | **C3(%)** | **A3(%)** | **G3(%)** | **AU(%)** | **GC(%)** | **GC3** | **GC12** | **ENC** | **GC3s** | **CAI** |
| --- | --- | --- | --- | --- | --- | --- | --- | --- | --- | --- | --- | --- | --- | --- | --- |
| **A (%)** |  |  |  |  |  |  |  |  |  |  |  |  |  |  |  |
| **C (%)** | 0.061 |  |  |  |  |  |  |  |  |  |  |  |  |  |  |
| **G (%)** | **-0.947***** | -0.163 |  |  |  |  |  |  |  |  |  |  |  |  |  |
| **U (%)** | 0.011 | **-0.899***** | -0.030 |  |  |  |  |  |  |  |  |  |  |  |  |
| **U3(%)** | 0.045 | **-0.904***** | -0.015 | **0.959***** |  |  |  |  |  |  |  |  |  |  |  |
| **C3(%)** | 0.365 | **0.869***** | -0.519 | **-0.626*** | **-0.702*** |  |  |  |  |  |  |  |  |  |  |
| **A3(%)** | **0.673*** | -0.377 | -0.487 | 0.235 | 0.292 | -0.172 |  |  |  |  |  |  |  |  |  |
| **G3(%)** | **-0.938***** | -0.106 | **0.938***** | -0.059 | -0.043 | -0.480 | -0.571 |  |  |  |  |  |  |  |  |
| **AU(%)** | 0.376 | **-0.839*** | -0.311 | **0.894***** | **0.899***** | -0.517 | 0.526 | -0.343 |  |  |  |  |  |  |  |
| **GC(%)** | -0.573 | **0.618*** | 0.516 | **-0.715*** | **-0.781*** | 0.293 | **-0.692*** | 0.535 | **-0.889***** |  |  |  |  |  |  |
| **GC3** | -0.415 | **0.586*** | 0.270 | -0.522 | **-0.620*** | 0.406 | **-0.850**** | 0.338 | **-0.696*** | **0.870***** |  |  |  |  |  |
| **GC12** | -0.480 | 0.228 | **0.620*** | -0.473 | -0.332 | -0.149 | 0.002 | 0.540 | -0.515 | 0.353 | -0.084 |  |  |  |  |
| **ENC** | **-0.838*** | 0.200 | **0.713*** | -0.125 | -0.225 | 0.011 | **-0.736*** | **0.632*** | -0.486 | **0.613*** | 0.527 | 0.299 |  |  |  |
| **GC3s** | **-0.903***** | 0.139 | **0.811*** | -0.187 | -0.270 | -0.165 | **-0.820*** | **0.870***** | -0.504 | **0.751*** | **0.679*** | 0.263 | **0.821*** |  |  |
| **CAI** | 0.050 | -0.310 | -0.034 | 0.199 | 0.113 | -0.243 | -0.038 | 0.009 | 0.168 | 0.148 | 0.311 | -0.448 | -0.269 | 0.058 |  |
| **Axis1** | -0.573 | **-0.586*** | **0.671*** | 0.326 | 0.406 | **-0.844**** | -0.100 | **0.720*** | 0.139 | 0.075 | -0.066 | 0.328 | 0.079 | 0.395 | 0.408 |
| **Axis2** | 0.231 | 0.543 | -0.059 | **-0.788*** | **-0.608*** | 0.243 | 0.178 | -0.071 | -0.546 | 0.317 | 0.073 | 0.566 | -0.290 | -0.189 | -0.162 |
| **Axis3** | -0.463 | **0.607**** | 0.339 | -0.498 | -0.565 | 0.429 | **-0.852**** | 0.289 | **-0.686*** | **0.794*** | **0.889***** | 0.058 | **0.717*** | **0.637*** | -0.022 |
| **Axis4** | -0.330 | 0.354 | 0.251 | -0.202 | -0.119 | 0.236 | -0.299 | 0.321 | -0.246 | 0.032 | -0.040 | 0.472 | 0.333 | 0.224 | **-0.773*** |

Note: *****p<0.0001; **p<0.001; *p<0.05**
