# Supplementary material for: Evolution of Codon Usage Bias in Henipaviruses Is Governed by Natural Selection and Is Host-Specific
Source: Viruses. 2018 Nov 1;10(11):604. doi: 10.3390/v10110604 (PMC6266499; doi:10.3390/v10110604)
Supplement: Supplementary file 1 [file viruses-10-00604-s001.zip › viruses-371287-supplementary/Supplementary Data/Supplementary Table 4.doc]

**Supplementary Table 4A:** Neutrality plot for entire coding sequences of NiV

| **NiV genes** | **Regression equations** | **Slopes Range** | **R^2^** | **P value** |
| --- | --- | --- | --- | --- |
| N | Y = 0.1471*X + 0.4239 | 0.1471 ± 0.1211 | 0.1019 | 0.2462 |
| P | Y = -0.3189*X + 0.5816 | -0.8053 to 0.1675 | 0.1337 | 0.1802 |
| W | Y = -0.2876*X + 0.6031 | -0.6339 to 0.05868 | 0.1984 | 0.0961 |
| V | Y = -0.1742*X + 0.5606 | -0.3863 to 0.03798 | 0.1948 | 0.0996 |
| C | Y = 0.5728*X + 0.05983 | 0.4417 to 0.7039 | 0.8726 | <0.0001 |
| M | Y = 0.2501*X + 0.2953 | -0.02106 to 0.5212 | 0.2339 | 0.0678 |
| F | Y = -0.08599*X + 0.4184 | -0.1418 to -0.03015 | 0.4598 | 0.0055 |
| G | Y = -0.2374*X + 0.4974 | -0.3325 to -0.1423 | 0.691 | 0.0001 |
| L | Y = -0.07617*X + 0.4135 | -0.1048 to -0.04758 | 0.7375 | <0.0001 |
| Whole Genome | Y = -0.05536*X + 0.4322 | -0.09594 to -0.01477 | 0.424 | 0.0117 |

**Supplementary Table 4B: Neutrality plot for entire coding sequences of HeV**

| **HeV genes** | **Regression equations** | **Slopes Range** | **R^2^** | **P value** |
| --- | --- | --- | --- | --- |
| N | Y = -0.04117*X + 0.5018 | -0.1311 to 0.04881 | 0.08441 | 0.219 |
| P | Y = 0.1136*X + 0.4187 | -0.1494 to 0.3765 | 0.07593 | 0.426 |
| W | Y = 0.3328*X + 0.3665 | 0.1351 to 0.5305 | 0.5551 | 0.014 |
| V | Y = 0.4215*X + 0.3369 | 0.1324 to 0.7107 | 0.4834 | 0.010 |
| C | Y = -0.1333*X + 0.4671 | -0.5158 to 0.2492 | 0.05079 | < 0.0001 |
| M | Y = 7.640e-007*X + 0.4081 | -0.07192 to 0.07192 | 4.97E-11 | 0.128 |
| F | Y = 0.01815*X + 0.3910 | -0.008536 to 0.04484 | 0.1693 | 0.1538 |
| G | Y = 0.05833*X + 0.3829 | -0.1522 to 0.2688 | 0.03271 | 0.365 |
| L | Y = 0.05913*X + 0.3684 | -0.08239 to 0.2006 | 0.07139 | 0.857 |
| Whole Genome | Y = 0.09760*X + 0.3765 | -0.04673 to 0.2419 | 0.1676 | 0.663 |
