# Supplementary material for: Evolution of Codon Usage Bias in Henipaviruses Is Governed by Natural Selection and Is Host-Specific
Source: Viruses. 2018 Nov 1;10(11):604. doi: 10.3390/v10110604 (PMC6266499; doi:10.3390/v10110604)
Supplement: Supplementary file 1 [file viruses-10-00604-s001.zip › viruses-371287-supplementary/Supplementary Data/Supplementary Table 5.doc]

**Supplementary Table 5A: The CAI values of different protein coding sequences of HeV in relation to various hosts.**

| **HeV genes** | ***Pteropus alecto*** | ***Pteropus vampyrus*** | ***Myotis brandtii*** | ***Myotis davidii*** | ***Myotis lucifugus*** | ***Eptesicus fuscus*** | ***Homo sapiens*** | ***Equus caballus*** | ***Sus scrofa*** | ***Canis familiaris*** | ***Felis catus*** |
| --- | --- | --- | --- | --- | --- | --- | --- | --- | --- | --- | --- |
| N | 0.762917 | 0.762333 | 0.753333 | 0.74125 | 0.749833 | 0.56175 | 0.731833 | 0.663 | 0.621 | 0.6665 | 0.66025 |
| P | 0.78575 | 0.784083 | 0.772917 | 0.760833 | 0.769833 | 0.584917 | 0.75125 | 0.677167 | 0.639167 | 0.68575 | 0.680417 |
| W | 0.78975 | 0.786083 | 0.775333 | 0.763333 | 0.771833 | 0.566 | 0.7535 | 0.683667 | 0.643333 | 0.6895 | 0.6835 |
| V | 0.79075 | 0.787083 | 0.77575 | 0.763333 | 0.772167 | 0.568833 | 0.753917 | 0.683667 | 0.642333 | 0.689 | 0.684833 |
| C | 0.771333 | 0.76825 | 0.761417 | 0.75325 | 0.76025 | 0.60725 | 0.74225 | 0.685083 | 0.655167 | 0.693167 | 0.696083 |
| M | 0.793 | 0.790167 | 0.77975 | 0.768833 | 0.77675 | 0.59425 | 0.761167 | 0.694583 | 0.65825 | 0.701167 | 0.694083 |
| F | 0.738417 | 0.735917 | 0.7235 | 0.7125 | 0.721583 | 0.563417 | 0.702583 | 0.625083 | 0.590333 | 0.635667 | 0.631917 |
| G | 0.771417 | 0.770667 | 0.757333 | 0.745333 | 0.754333 | 0.584833 | 0.736333 | 0.6595 | 0.618583 | 0.666667 | 0.659417 |
| L | 0.759917 | 0.754333 | 0.739917 | 0.72725 | 0.737167 | 0.583667 | 0.718167 | 0.636833 | 0.596083 | 0.645167 | 0.640917 |
| Complete | 0.773694 | 0.770991 | 0.759917 | 0.748435 | 0.757083 | 0.579435 | 0.739 | 0.66762 | 0.629361 | 0.674731 | 0.670157 |

**Supplementary Table 5B: The CAI values of different protein coding sequences of Ni**V in relation to various hosts.

| **NiV genes** | ***Pteropus alecto*** | ***Pteropus vampyrus*** | ***Myotis brandtii*** | ***Myotis davidii*** | ***Myotis lucifugus*** | ***Eptesicus fuscus*** | ***Homo sapiens*** | ***Equus caballus*** | ***Sus scrofa*** | ***Canis familiaris*** | ***Felis catus*** |
| --- | --- | --- | --- | --- | --- | --- | --- | --- | --- | --- | --- |
| N | 0.753667 | 0.752667 | 0.744 | 0.7314 | 0.739667 | 0.564467 | 0.721467 | 0.650933 | 0.6088 | 0.654667 | 0.6492 |
| P | 0.7734 | 0.771867 | 0.760067 | 0.747667 | 0.757667 | 0.578533 | 0.7378 | 0.660467 | 0.6206 | 0.668267 | 0.667667 |
| W | 0.782467 | 0.780867 | 0.7708 | 0.757867 | 0.7666 | 0.575733 | 0.747133 | 0.6724 | 0.630667 | 0.6792 | 0.678267 |
| V | 0.7766 | 0.775 | 0.764267 | 0.751267 | 0.760733 | 0.5692 | 0.740733 | 0.6666 | 0.624333 | 0.673 | 0.674 |
| C | 0.782467 | 0.7826 | 0.774133 | 0.765133 | 0.772133 | 0.6248 | 0.755067 | 0.692867 | 0.6604 | 0.700733 | 0.699267 |
| M | 0.7842 | 0.786467 | 0.775467 | 0.764267 | 0.7722 | 0.588933 | 0.755867 | 0.687667 | 0.6478 | 0.6922 | 0.686467 |
| F | 0.7418 | 0.7368 | 0.723267 | 0.7118 | 0.721133 | 0.562867 | 0.7008 | 0.617133 | 0.581067 | 0.628467 | 0.623733 |
| G | 0.7622 | 0.7592 | 0.745 | 0.733133 | 0.742667 | 0.569733 | 0.7234 | 0.644733 | 0.6044 | 0.652933 | 0.649067 |
| L | 0.755 | 0.749429 | 0.734714 | 0.722643 | 0.732429 | 0.582429 | 0.713357 | 0.632 | 0.592071 | 0.639929 | 0.635929 |
| Complete | 0.767978 | 0.766099 | 0.754635 | 0.742797 | 0.751692 | 0.579633 | 0.732847 | 0.658311 | 0.618904 | 0.665488 | 0.662622 |
