# Supplementary material for: Evolution of Codon Usage Bias in Henipaviruses Is Governed by Natural Selection and Is Host-Specific
Source: Viruses. 2018 Nov 1;10(11):604. doi: 10.3390/v10110604 (PMC6266499; doi:10.3390/v10110604)
Supplement: Supplementary file 1 [file viruses-10-00604-s001.zip › viruses-371287-supplementary/Supplementary Data/Supplementary Table 6.doc]

**Supplementary Table 6: The tRNA adaptation index for NiV isolates.**

| **NiV Isolates** | **tIA** | |
| --- | --- | --- |
| **Malaysia Isolates** | **W** | **G** |
| AF376747 Nipah virus Malaysia nucleocapsid protein | 0.319765 | 0.384133 |
| AJ627196 Nipah virus NV/MY/99/VRI-0626 nucleocapsid protein | 0.320213 | 0.384999 |
| AJ564623 Nipah virus NV/MY/99/UM-0128 nucleocapsid protein | 0.319645 | 0.384133 |
| AJ564622 Nipah virus NV/MY/99/VRI-1413 nucleocapsid protein | 0.319645 | 0.384197 |
| AJ564621 Nipah virus NV/MY/99/VRI-2794 nucleocapsid protein | 0.319645 | 0.384133 |
| AY029768 Nipah virus UMMC2 nucleocapsid protein | 0.319645 | 0.384197 |
| AY029767 Nipah virus UMMC1 nucleocapsid protein | 0.319645 | 0.384197 |
| **Bangladesh/India Isolates** | | |
| AY988601 Nipah virus Bangladesh nucleocapsid protein | 0.32272 | 0.395735 |
| JN808863 Nipah virus NIVBGD2008RAJBARI nucleocapsid protein | 0.325661 | 0.394445 |
| JN808864 Nipah virus NIVBGD2010FARIDPUR nucleocapsid protein | 0.324453 | 0.397082 |
| JN808857 Nipah virus NIVBGD2008MANIKGONJ nucleocapsid protein | 0.325661 | 0.394445 |
| FJ513078 Nipah virus Ind-Nipah-07-FG nucleocapsid protein | 0.324552 | 0.394504 |
